# Tandem occlusions involving the internal carotid and anterior cerebral arteries—A rare form of stroke: Results from the multicenter EVATRISP collaboration study

**DOI:** 10.3389/fneur.2022.1024891

**Published:** 2022-12-09

**Authors:** Andrei Filioglo, Naaem Simaan, Asaf Honig, Mirjam Heldner, Alessandro Pezzini, Nicolas Martinez-Majander, Visnja Padjen, Philipp Baumgartner, Panagiotis Papanagiotou, Alexander Salerno, Christian Nolte, Annika Nordanstig, Stefan Engelter, Andrea Zini, Marialuisa Zedde, João Pedro Marto, Marcel Arnold, Mauro Magoni, Henrik Gensicke, Jose Cohen, Ronen Leker

**Affiliations:** ^1^Department of Neurology, Hadassah-Hebrew University Medical Center, Jerusalem, Israel; ^2^Department of Neurology, University Hospital Bern, Bern, Switzerland; ^3^Department of Clinical and Experimental Sciences, University of Brescia, ASST Spedali Civili, Brescia, Italy; ^4^Department of Neurology, Helsinki University Hospital and Clinical Neurosciences, University of Helsinki, Helsinki, Finland; ^5^Faculty of Medicine, Neurology Clinic, University Clinical Centre of Serbia, University of Belgrade, Belgrade, Serbia; ^6^University Hospital Zurich, University of Zurich, Zurich, Switzerland; ^7^Department of Diagnostic and Interventional Neuroradiology, Hospital Bremen-Mitte/Bremen-Ost, Bremen, Germany; ^8^Stroke Center, Neurology Service, Department of Clinical Neurosciences, Lausanne University Hospital, University of Lausanne, Lausanne, Switzerland; ^9^Department of Neurology, Charité-Universitätsmedizin Berlin, Berlin, Germany; ^10^Center for Stroke Research, Berlin Institute of Health, Berlin, Germany; ^11^Department of Clinical Neurosciences, Institute of Neurosciences and Physiology, Sahlgrenska Academy at University of Gothenburg, Gothenburg, Sweden; ^12^Department of Neurology, Sahlgrenska University Hospital, Gothenburg, Sweden; ^13^Stroke Center and Department of Neurology, University Hospital Basel, University of Basel, Basel, Switzerland; ^14^Department of Neurology and Stroke Center, IRCCS Istituto delle Scienze Neurologiche di Bologna, Maggiore Hospital, Bologna, Italy; ^15^Neurology Unit, Stroke Unit, Azienda Unità Sanitaria Locale-IRCCS di Reggio Emilia, Reggio Emilia, Italy; ^16^Department of Neurology, Hospital de Egas Moniz, Centro Hospitalar Lisboa Ocidental, Lisbon, Portugal; ^17^Department of Neurology and Neurorehabilitation, University Department of Geriatric Medicine FELIX PLATTER, University of Basel, Basel, Switzerland; ^18^Department of Neurosurgery, Hadassah-Hebrew University Medical Center, Jerusalem, Israel

**Keywords:** cerebrovascular disease, endovascular, stroke, thrombectomy, anterior cerebral artery

## Abstract

**Background:**

Patients with stroke secondary to isolated anterior cerebral artery (ACA) occlusions have poor outcomes. Whether tandem occlusions (TO) of the extracranial internal carotid (ICA) and the ACA carry even worse outcomes that remain unknown.

**Methods:**

Patients with TO involving ICA and ACA occlusions were identified from 14 participating centers from the EndoVascular treatment And ThRombolysis in Ischemic Stroke Patients (EVATRISP) project which is a multicenter, observational, cohort study with prospective accrual of data followed by retrospective data analysis. Patients with isolated ACA stroke served as controls.

**Results:**

Included were 92 patients with isolated ACA and 16 patients with ICA-ACA TO stroke. On univariate analyses, patients with TO had more severe strokes on admission [median NIHSS (IQR) 13.5 (9–21) vs. 8 (5–12), *p* = 0.003] and were more often treated with thrombectomy (81 vs. 40%, *p* = 0.002). Mortality rates were higher among TO patients (31 vs. 11%, *p* = 0.03). Rates of favorable functional outcomes were numerically lower among TO patients (38 vs. 60%) but the difference was not statistically significant (*p* = 0.09). On multivariate analyses, the presence of TO did not modify the chances for favorable outcomes.

**Conclusion:**

TO stroke with ICA and isolated ACA involvement is rare and results in more severe initial neurological deficits and higher mortality compared to those seen in patients with isolated ACA stroke.

## Introduction

Tandem large vessel occlusions (TO) involving the extracranial internal carotid artery (ICA) and various intracranial arteries account for 13–30% of all large vessel occlusions (LVO) (1–4) and are associated with poor outcomes (3, 5). Endovascular thrombectomy (EVT) is the treatment of choice for TO with or without intravenous thrombolysis (IVT) and ICA stenting (CAS) (1–8).

Most TO involve the ICA and the middle cerebral artery (MCA) with involvement of anterior cerebral artery (ACA) being rarely observed (3). Isolated ACA stroke is associated with severe stroke and high rates of poor functional outcomes (9). Whether this becomes even worse when ACA involvement is part of TO remains unknown. Therefore, we aimed to evaluate the outcomes of patients with TO involving ACA occlusions in comparison with isolated occlusions of the ACA.

## Materials and methods

### Study design and population

For the current study, we used pooled data from 14 centers participating in the EVATRISP (The EndoVascular treatment And ThRombolysis in Ischemic Stroke Patients), international multicenter collaboration (10). EVATRISP is a multicenter, observational, cohort study with prospective accrual of data followed by retrospective data analysis.

The studies involving human participants were reviewed and approved by the institutional review board of the Hadassah Medical Organization with an exemption from obtaining individual informed consent forms due to the anonymized nature of data collection and the retrospective nature of the study (Approval # HMO-0378-18). Similarly, the study was approved by all participating centers.

Patients with TO secondary to ICA and ACA occlusions who underwent IVT, EVT, or bridging therapy between 3/2015 and 7/2020 were included. TO was diagnosed in patients with extracranial ICA occlusions or extracranial bifurcation ICA stenosis >70% measured on CT angiography maximal intensity projection sections in sagittal, coronal, and axial planes. The anatomy of the circle of Willis was studied on maximal intensity projections CT angiography reconstruction in order to detect anatomical variants and anomalies. Patients with isolated ACA occlusions during the same time epoch were recruited from the same databases and served as controls. TO secondary to intracranial ICA-T occlusions were excluded, as were those with ICA and ACA lesions that also had concomitant middle cerebral artery (MCA) involvement.

Clinical and radiological data were prospectively recorded based on a standardized electronic data collection form that was used in all participating centers as previously detailed (10). Local institutional review boards approved the study in each of the participating centers.

Acute symptomatic ICA treatment was defined as treatment of the extracranial ICA lesion with either stenting or balloon angioplasty alone at the time of EVT. Data on procedural details including primary and rescue endovascular strategies, number of passes, intra-arterial injection of glycoprotein IIb/IIIa antagonists, procedure complications, reasons for EVT failure, and recanalization score were analyzed with the modified thrombolysis in cerebral infarction (mTICI) (11) scale with mTICI 2b-3 considered as successful recanalization and mTICI 3 as excellent recanalization.

EVT strategy was chosen according to interventionists' judgment. Bridging with intravenous alteplase 0.9 mg/kg prior to EVT was allowed.

Intracranial hemorrhage (ICH) was classified according to European Cooperative Acute Stroke Study (ECASS) II criteria (12). Favorable functional outcome was defined as functional independence at 90-day post-stroke [modified Rankin Scale (mRS) 0–2 or equal to the pre-stroke mRS score for patients with a pre-stroke mRS score of 3 or higher] (13). Mortality was defined as death occurring within 90 days.

The cohort of TO ACA patients was further analyzed in an attempt to identify prognostic factors associated with outcome in this group of patients.

Statistical analyses were performed using the SPSS 25 (IBM USA). The χ^2^ test was used to explore the link between dichotomous variables. The Student's t-test was used to compare means and non-parametric tests were used to compare medians. Variables with a *p* < 0.10 on univariate analysis were included in multivariate logistic regression models determining the adjusted odds ratio (OR) for the outcomes of favorable outcome.

## Results

The EVATRISP database includes information on over 10,000 patients treated with EVT (±tPA). Throughout the study period, 16 patients with TO involving the ICA and ACA were identified in the 14 participating centers. During the study epoch, 873 patients with tandem ICA-MCA occlusions were treated at the participating centers for a ratio of 1:55. Among the TO patients, 12 (75%) had extracranial ICA occlusions and 4 had severe ICA stenosis (≥70%). Five patients received acute stenting as part of EVT for the acute stroke. This involved an anterograde approach in 4/5 with stenting the ICA first followed by thrombectomy of the ACA. Evaluation of the circle of Willis anatomy at the participating centers revealed an acute exit angle of the ipsilateral MCA in five of the 16 patients and a proximal ipsilateral MCA stenosis in another patient.

Compared to the 92 patients with isolated intracranial ACA occlusions identified during the same epoch (Table 1), patients with TO had more severe neurological deficits at admission [median NIHSS (IQR) 13.5 (9–21.5) vs. 8 (5–12), *p* = 0.003] and were more often treated with thrombectomy (81 vs. 40%, *p* = 0.002). Trends toward higher rates of sICH were also observed, but did not reach statistical significance (Table 1). Mortality rates were higher among TO patients (31 vs. 11%, *p* = 0.03). Rate of favorable functional outcome was numerically lower among TO patients (38 vs. 60%) but the difference was not statistically significant (*p* = 0.09). Factors suggesting association with favorable outcomes among the entire cohort (Table 2) included younger age, smoking, lower admission NIHSS, and higher improvement in stroke severity (ΔNIHSS) from admission to discharge, and treatment modality. Only 3 of the TO patients were given IVT mainly because of late presentation or prior use of anticoagulants. Patients treated with IVT either alone or as part of a bridging protocol had higher chances of favorable outcomes, whereas clot location within the ACA had no effect on the likelihood of favorable functional outcome.

**Table 1:** Characteristics of patients with ACA stroke.

**Characteristics**	**Isolated ACA (*N* = 92)**	**TO ACA (*N* = 16)**	***P***
Age (median, IQR)	74 (64–82.4)	69 (56–78.3)	0.847
Sex male (%)	50 (54)	9 (56)	0.888
Hypertension (%)	71 (77)	14 (88)	0.352
Diabetes (%)	21 (23)	4 (25)	0.849
Atrial fibrillation (%)	30 (33)	3 (19)	0.267
Cholesterol (%)	50 (54)	13 (81)	0.044
Smoking (%)	18 (20)	4 (29)	0.452
Ischemic heart disease (%)	19 (21)	2 (13)	0.447
Prior stroke (%)	24 (26)	3 (19)	0.532
Wake-up stroke (%)	10 (15)	3 (19)	0.743
Transfer (%)	12 (19)	4 (25)	0.576
admission NIHSS (median, IQR)	8 (5–12)	13.5 (9–21.5)	0.003
Treatment modality			0.002
IVT (%)	55 (60)	3 (19)	
EVT (%)	37 (40)	13 (81)	
ACA occlusion site (%)			0.338
A1	29 (32)	7 (44)	
A2/A3	63 (68)	9 (56)	
Intra-arterial lytics (%)	6 (15)	3 (19)	0.759
Complete recanalization (%)	25 (64)	6 (46)	0.253
Symptomatic ICH (%)	2 (3)	2 (13)	0.068
In-hospital complications (%)	31 (43)	9 (56)	0.315
Length of admission (median, IQR)	7 (4–11)	4 (3–11)	0.210
Δ NIHSS (median, IQR)	3 (1.25–7)	2.5 (−14 to 10)	0.974
Favorable outcome (%)	55 (60)	6 (38)	0.097
Mortality (%)	10 (11)	5 (31)	0.030

ACA, anterior cerebral artery; IVT, intravenous thrombolysis; EVT, endovascular thrombectomy; NIHSS, NIH stroke scale; ICH, intracerebral hemorrhage.

**Table 2:** Outcomes in patients with ACA stroke and tandem occlusions.

**Characteristics**	**Favorable outcome**	**Unfavorable outcome**	***p***
	***N* = 61**	***N* = 47**	
Age (median, IQR)	72 (54.5–80.75)	78 (72–85.5)	0.020
Sex male (%)	37 (61)	22 (47)	0.152
Hypertension (%)	48 (79)	37 (79)	0.996
Diabetes (%)	12 (20)	13 (28)	0.329
Atrial fibrillation (%)	16 (26)	17 (36)	0.266
Cholesterol (%)	33 (54)	30 (64)	0.309
Smoking (%)	18 (30)	4 (9)	0.011
Ischemic heart disease (%)	13 (21)	8 (17)	0.576
Prior stroke (%)	13 (21)	14 (30)	0.313
Wake-up stroke (%)	6 (15)	7 (17)	0.799
Transfer (%)	6 (15)	10 (24)	0.314
Admission NIHSS (median, IQR)	7 (5–11)	10 (7.7–16)	0.002
Treatment modality			0.005
IVT (%)	40 (66)	18 (38)	
EVT (%)	21 (34)	29 (62)	
ACA occlusion site (%)			0.170
A1	17 (28)	19 (40)	
A2/A3	44 (72)	28 (60)	
Tandem occlusion (%)	6 (10)	10 (21)	0.097
Intra-arterial lytics (%)	2 (8)	7 (23)	0.126
Complete recanalization (%)	15 (68)	16 (53)	0.281
Symptomatic ICH (%)	0 (0)	4 (10)	0.021
In-hospital complications (%)	16 (35)	24 (56)	0.046
Length of admission (median, IQR)	5 (4–10)	7 (3–12.5)	0.289
Δ NIHSS (median, IQR)	4 (3–7)	1 (−2 to 4.5)	0.001

ACA, anterior cerebral artery; IVT, intravenous thrombolysis; EVT, endovascular thrombectomy; NIHSS, NIH Stroke Scale; ICH, intracerebral hemorrhage.

On multivariate regression analyses (Table 3), age (OR 0.94, 95% CI 0.89–0.98), stroke severity (OR 0.74, 95% CI 0.64–0.87), and change in NIHSS from baseline to discharge (OR 1.36, 95% CI 1.16–1.59) were associated with favorable outcome, whereas the presence of TO (OR 0.77, 95% CI 0.11–5.46) was not.

**Table 3:** Multivariate analysis for favorable outcome.

	**OR**	**95% CI**		***P***
Age	0.938	0.894	0.985	0.011
Tandem occlusion	0.769	0.108	5.461	0.793
NIHSS upon admission	0.743	0.639	0.864	< 0.001
Treatment modality	0.707	0.223	2.246	0.557
Delta NIHSS	1.361	1.164	1.591	< 0.001

NIHSS, NIH stroke scale.

We further attempted to identify prognostic markers among patients with TO (Supplementary Table 1). In this group of 16 patients, the only factors that were associated with increased rates of favorable outcome were lower initial stroke severity, treatment with IVT and a larger change in NIHSS from admission to discharge. Given the very small number of patients in this group regression analyses could not be performed.

## Discussion

The main findings of the current study are that TO involving the ICA and ACA is a rare phenomenon that is associated with higher stroke severity and results in higher mortality rates, compared with isolated ACA stroke.

The rarity of this form of TO is immediately apparent as only 16 patients were identified in our prospective registry of 14 academic centers over the span of 5 years compared to 873 patients with ICA-MCA TO treated during the same period for a ratio of 1:55. Indeed, most previous studies report on TO involving either ICA and MCA or ICA and multiple territorial infarcts but not isolated ICA-ACA TO stroke. This could potentially be explained, by the fact that the MCA has a larger diameter and can be considered to be in direct continuation to the ICA, whereas the ACA usually comes off the ICA at an angle and is smaller in caliber, making embolic material from the extracranial ICA to preferentially migrate to the MCA (14). In the current series, 5/16 patients had an acute angle of exit of the MCA from the circle of Willis, which could theoretically account for emboli migration into the ACA. Notably, circle of Willis geometry has been associated with stroke risk (15, 16); and even bi-hemispheric ACA strokes have been shown to originate from a single carotid source (17). Alternatively, focal stenosis of the proximal M1 MCA segment may increase luminal pressure in the MCA diverting clots to the ACA. This phenomenon was seen in 1/16 patients with TO-ACA in the current series. This would imply that in the remaining 10/16 patients emboli migration to the ACA was caused by another mechanism or was a chance finding. The absence of a centralized core imaging laboratory and the use of different CT angiography techniques and projections in different participating centers precluded us from further testing these hypotheses.

Therapeutic options in ACA occlusion include IVT and/or EVT and equipoise exists as to the best treatment option in the absence of randomized data (14, 18–20). Interestingly, treatment with IVT was a marker of favorable outcome in the univariate analysis in our entire cohort comprising of both TO and isolated ACA stroke as well as in the TO patients only. Previous studies reported higher recanalization rates among patients with ICA-MCA TO stroke treated with IV lytics in combination with endovascular techniques supporting the validity of the current findings (3, 7, 13). However, this association did not persist after controlling for age and stroke severity in the multivariate regression analyses probably due to the low number of included patients. Similarly, the relatively low number of ICA-ACA TO patients identified led to our inability to show effects of this stroke subtype on functional outcome. Importantly, our results show that successful target vessel recanalization rates were similar for the TO and isolated ACA stroke groups in the EVT-treated patients and this could likely contribute to the lack of statistical difference in functional outcomes because recanalization is a powerful determinant of outcome. Similar results were observed in the ESCAPE study looking at ICA-MCA TO where successful recanalization was associated with increased likelihood of favorable outcomes (2). Unfortunately, due to the very small number of included TO patients, we could not assess the impact of procedure-related variables such as technique (e.g., stent first vs. thrombectomy first), number of passes needed or types of devices used (2–4, 6, 7, 21). Therefore, we suggest that future larger prospective registries exploring these parameters should be launched.

The strength of the current study is that it represents an international multicenter cohort of prospectively enrolled patients and thus accurately reflects real-world data.

The limitations of the current study are immanent to registry data including potential selection bias and bias by indication. However, the rarity of ICA-ACA TO precludes a randomized-controlled study in our opinion. Another limitation pertains to the low number of included patients which risks type 2 error in our univariate and overfitting in the multivariate regression analyses. Therefore, our data should be interpreted with caution.

In conclusion, our results suggest that TO involving the ICA and ACA is a rare form of stroke, characterized by higher initial stroke severity and higher mortality rates compared to isolated ACA stroke. Future prospective multicentric registry data are needed to better characterize this rare form of stroke and to determine whether different subgroups of patients with ICA-ACA TO can benefit from different therapeutic strategies such as direct EVT vs. bridging with IVT as well as specific endovascular approaches (i.e., clot aspiration vs. stentriever-based thrombectomy vs. combined approach).

## Data availability statement

The raw data supporting the conclusions of this article will be made available by the authors, without undue reservation.

## Ethics statement

The studies involving human participants were reviewed and approved by the Institutional Review Board of the Hadassah Medical Organization (Approval# HMO-0378-18). Written informed consent from the patients/participants or patients/participants' legal guardian/next of kin was not required to participate in this study in accordance with the national legislation and the institutional requirements.

## Author contributions

AF, NS, and RL designed the work including data acquisition, analysis, and drafting of the manuscript. AH, MH, AP, NM-M, VP, PB, PP, AS, CN, AN, SE, AZ, MZ, JM, MA, MM, HG, and JC took part in manuscript revision and further analysis. All authors contributed to the article and approved the submitted version.
