# Corrigendum: Tandem occlusions involving the internal carotid and anterior cerebral arteries—A rare form of stroke: results from the multicenter EVATRISP collaboration study

**DOI:** 10.3389/fneur.2024.1450372

**Published:** 2024-07-01

**Authors:** Andrei Filioglo, Naaem Simaan, Asaf Honig, Mirjam Heldner, Alessandro Pezzini, Nicolas Martinez-Majander, Visnja Padjen, Philipp Baumgartner, Panagiotis Papanagiotou, Alexander Salerno, Christian Nolte, Annika Nordanstig, Stefan Engelter, Andrea Zini, Marialuisa Zedde, João Pedro Marto, Marcel Arnold, Mauro Magoni, Henrik Gensicke, Jose Cohen, Ronen Leker

**Affiliations:** ^1^Department of Neurology, Hadassah-Hebrew University Medical Center, Jerusalem, Israel; ^2^Department of Neurology, University Hospital Bern, Bern, Switzerland; ^3^Department of Clinical and Experimental Sciences, University of Brescia, ASST Spedali Civili, Brescia, Italy; ^4^Department of Neurology, Helsinki University Hospital and Clinical Neurosciences, University of Helsinki, Helsinki, Finland; ^5^Faculty of Medicine, Neurology Clinic, University Clinical Centre of Serbia, University of Belgrade, Belgrade, Serbia; ^6^University Hospital Zurich, University of Zurich, Zurich, Switzerland; ^7^Department of Diagnostic and Interventional Neuroradiology, Hospital Bremen-Mitte/Bremen-Ost, Bremen, Germany; ^8^Stroke Center, Neurology Service, Department of Clinical Neurosciences, Lausanne University Hospital, University of Lausanne, Lausanne, Switzerland; ^9^Department of Neurology, Charité-Universitätsmedizin Berlin, Berlin, Germany; ^10^Center for Stroke Research, Berlin Institute of Health, Berlin, Germany; ^11^Department of Clinical Neurosciences, Institute of Neurosciences and Physiology, Sahlgrenska Academy at University of Gothenburg, Gothenburg, Sweden; ^12^Department of Neurology, Sahlgrenska University Hospital, Gothenburg, Sweden; ^13^Stroke Center and Department of Neurology, University Hospital Basel, University of Basel, Basel, Switzerland; ^14^Department of Neurology and Stroke Center, IRCCS Istituto delle Scienze Neurologiche di Bologna, Maggiore Hospital, Bologna, Italy; ^15^Neurology Unit, Stroke Unit, Azienda Unità Sanitaria Locale-IRCCS di Reggio Emilia, Reggio Emilia, Italy; ^16^Department of Neurology, Hospital de Egas Moniz, Centro Hospitalar Lisboa Ocidental, Lisbon, Portugal; ^17^Department of Neurology and Neurorehabilitation, University Department of Geriatric Medicine FELIX PLATTER, University of Basel, Basel, Switzerland; ^18^Department of Neurosurgery, Hadassah-Hebrew University Medical Center, Jerusalem, Israel

**Keywords:** cerebrovascular disease, endovascular, stroke, thrombectomy, anterior cerebral artery

In the published article, the reference for (17) was incorrectly written as Strambo D. Acute bihemispheric stroke from a single carotid source: risk factors, mechanism and outcome. *J Vasc Interv Neurol*. (2021) 12:24–33. It should be Scoppettuolo P, Strambo D, Nannoni S, Dunet V, Sirimarco G, Michel P. Acute bihemispheric stroke from a single carotid source: risk factors, mechanism and outcome. *J Vasc Interv Neurol*. (2021) 12:24–33. 10.5281/zenodo.10391282.

The authors apologize for this error and state that this does not change the scientific conclusions of the article in any way. The original article has been updated.

## References

[17] Scoppettuolo P Strambo D Nannoni S Dunet V Sirimarco G Michel P. Acute bihemispheric stroke from a single carotid source: risk factors, mechanism and outcome. J Vasc Interv Neurol. (2021) 12:24–33. 10.5281/zenodo.10391282

