# Supplementary material for: Tandem occlusions involving the internal carotid and anterior cerebral arteries—A rare form of stroke: Results from the multicenter EVATRISP collaboration study
Source: Front Neurol. 2022 Dec 9;13:1024891. doi: 10.3389/fneur.2022.1024891 (PMC9780389; doi:10.3389/fneur.2022.1024891)
Supplement: Supplementary file 1 [file Table_1.DOCX]

| Characteristics | Favorable outcome  N=6 | Unfavorable outcome  N=10 | P |
| --- | --- | --- | --- |
| Age (SD) | 61.8 (15.6) | 70.4 (10.7) | 0.208 |
| Sex male (%) | 2 (33) | 7 (70) | 0.152 |
| Hypertension (%) | 6 (100) | 8 (80) | 0.242 |
| Diabetes (%) | 2 (33) | 2 (20) | 0.551 |
| Atrial fibrillation (%) | 1 (17) | 2 (20) | 0.869 |
| Cholesterol (%) | 4 (67) | 9 (90) | 0.247 |
| Smoking (%) | 3 (50) | 1 (10) | 0.124 |
| Ischemic heart disease (%) | 1 (17) | 1 (10) | 0.696 |
| Prior stroke (%) | 1 (17) | 2 (20) | 0.869 |
| Wake-up stroke (%) | 2 (33) | 1 (10) | 0.247 |
| Transfer (%) | 1 (17) | 3 (30) | 0.551 |
| admission NIHSS (SD) | 10 (7) | 20 (10) | 0.042 |
| Extracranial ICA stenosis (%) |  |  | 0.491 |
| 50-70% | 1 (17) | 1 (10) |  |
| 70-99% | 0 (0) | 2 (20) |  |
| Occlusion | 5 (83) | 7 (70) |  |
| Treatment modality (%) |  |  | 0.013 |
| IVT (%) | 3 (50) | 0 (0) |  |
| EVT (including bridging) (%) | 3 (50) | 10 (100) |  |
| Acute CAS (%) | 2 (33) | 3 (30) | 0.889 |
| Intra-arterial lytics (%) | 0 (0) | 3 (30) | 0.137 |
| Complete recanalization (%) | 2 (67) | 4 (40) | 0.416 |
| Symptomatic ICH (%) | 0 (0) | 2 (20) | 0.242 |
| In- hospital complications (%) | 2 (33) | 7 (70) | 0.152 |
| Delta NIHSS, (SD) | 7 (4) | -7 (13) | 0.025 |
| Length of admission (SD) | 6.5 (4) | 10.7 (16) | 0.549 |

Supplementary Table 1: Comparison of outcomes among patients with ICA-ACA tandem occlusions
